# Oxidative Stress, Endoparasite Prevalence and Social Immunity in Bee Colonies Kept Traditionally vs. Those Kept for Commercial Purposes

**DOI:** 10.3390/insects11050266

**Published:** 2020-04-27

**Authors:** Elmin Taric, Uros Glavinic, Branislav Vejnovic, Aleksandar Stanojkovic, Nevenka Aleksic, Vladimir Dimitrijevic, Zoran Stanimirovic

**Affiliations:** 1 Department of Biology, Faculty of Veterinary Medicine, University of Belgrade, Bulevar oslobodjenja 18, 11000 Belgrade, Serbia; etar1989@yahoo.com (E.T.); zoran@vet.bg.ac.rs (Z.S.); 2 Department of Economics and Statistics, Faculty of Veterinary Medicine, University of Belgrade, Bulevar oslobodjenja 18, 11000 Belgrade, Serbia; branislavv@vet.bg.ac.rs; 3 Department of Animal Source Foods Science and Technology, Institute for Animal Husbandry, Autoput 16, 11080 Belgrade–Zemun, Serbia; vetalex@gmx.com; 4 Department of Parasitology, Faculty of Veterinary Medicine, University of Belgrade, Bulevar oslobodjenja 18, 11000 Belgrade, Serbia; nenaaleksic@vet.bg.ac.rs; 5 Department of Animal Husbandry and Genetics, Faculty of Veterinary Medicine, University of Belgrade, Bulevar oslobodjenja 18, 11000 Belgrade, Serbia; vanja@vet.bg.ac.rs

**Keywords:** *Apis mellifera*, *Lotmaria passim*, *Nosema ceranae*, commercial beekeeping, traditional beekeeping

## Abstract

Commercially and traditionally managed bees were compared for oxidative stress (superoxide dismutase (SOD), catalase (CAT), glutathione S-transferase (GST) and malondialdehyde (MDA)), the prevalence of parasites (*Lotmaria passim*, *Crithidia mellificae* and *Nosema ceranae/apis*) and social immunity (glucose oxidase gene expression). The research was conducted on Pester plateau (Serbia—the Balkan Peninsula), on seemingly healthy colonies. Significant differences in CAT, GST and SOD activities (*p* < 0.01), and MDA concentrations (*p* < 0.002) were detected between commercial and traditional colonies. In the former, the prevalence of both *L. passim* and *N. ceranae* was significantly (*p* < 0.05 and *p* < 0.01, respectively) higher. For the first time, *L. passim* was detected in honey bee brood. In commercial colonies, the prevalence of *L. passim* was significantly (*p* < 0.01) lower in brood than in adult bees, whilst in traditionally kept colonies the prevalence in adult bees and brood did not differ significantly. In commercially kept colonies, the GOX gene expression level was significantly (*p* < 0.01) higher, which probably results from their increased need to strengthen their social immunity. Commercially kept colonies were under higher oxidative stress, had higher parasite burdens and higher GOX gene transcript levels. It may be assumed that anthropogenic influence contributed to these differences, but further investigations are necessary to confirm that.

## 1. Introduction

It has recently been shown that commercially kept *Apis mellifera* Linnaeus, 1758 colonies in standard hives are more burdened both with bee brood pathogens (*Paenibacillus larvae*, *Melissococcus plutonius*, *Ascosphaera apis* and sacbrood virus—SBV) and adult bee pathogens (deformed wing virus—DWV, chronic bee paralysis virus—CBPV and acute bee paralysis virus—ABPV) than their counterparts kept traditionally in so-called trmka hives [1]. There was other research on health status and pathogen loads of feral vs. commercial (managed) colonies [2,3,4,5] but the results are not consistent. 

It is considered that the primary response of bees to infection is the production of reactive oxygen species (ROS), which are produced in physiological processes and take part in defence against infective agents. Unfortunately, ROS are incapable of distinguishing between the hosts’ and the pathogens’ macromolecules [6]. However, in cases of hyperproduction of ROS or decreased antioxidative defence, a condition known as oxidative stress develops. Increased production of free radicals may do damage to proteins, membrane lipids and nucleic acids [7]. Given that the values of the oxidative stress parameters may significantly change due to the invasion of pathogens, their assessment may be a useful marker of the efficacy of defence mechanisms [8,9]. It is to be emphasised that there are few papers on the oxidative profile of bees infected with pathogens [9,10].

The immune system is one of the animals’ most expensive physiological systems to maintain, especially when food is deficient in proteins [2,11,12], which is extremely frequent in commercially kept colonies, usually fed on sugar syrup, in contrast to traditional bee colonies fed exclusively on natural feed [1]. The glucose oxidase (GOX) enzyme is directly connected with diet: colonies which originate from polyfloral areas have increased activities of GOX in comparison with the bees feeding on a monofloral diet [11]. Insects have a powerful immune system, which fights against the attack of various pathogens [13]. GOX was used as a parameter of social immunity [14,15] owing to the fact that it enables bees to sterilise brood food (honey and bee bread) and the colony itself via the by-product of GOX, d-gluconic acid and hydrogen peroxide (H_2_O_2_) [16,17,18], although recent findings revealed no increase in GOX gene expression neither after exposure to the microsporidian and a neonicotinoid pesticide [14], nor following the challenge with a bacterial pathogen [19]. 

Nosemosis is a disease of adult bees which is caused by intracellular parasites *Nosema apis* Zander, 1909 and *N. ceranae* Fries, 1996 [20,21]. It has been detected that *N. ceranae* increases energetic stress in bees [22,23], has an immunosuppressive effect [24,25,26] and shortens the life span of infected bees [10,27,28]. Two trypanosoma species, *Lotmaria passim* Schwarz, 2014 and *Crithidia mellificae* Langridge and McGhee, 1967, have recently been considered to be possible causes of bee decline, which is why they are to be studied in order to better understand the population dynamics of this phenomenon [5]. 

Following the initial description of *L. passim* [29] and the establishment of molecular methods for detection and distinguishing between *C. mellificae* and *L. passim*, several surveys were accomplished, which all confirmed the results by Schwarz et al. [29], who claimed that *L. passim* is the dominant trypanosomatid species in *A. mellifera* [5,30,31,32]. 

In a retrospective analysis of samples collected in Serbia in a nine-year period (2007–2015), a high prevalence (60.5%) of coinfection with *L. passim* and *N. ceranae* [32] was detected. The relation between Nosema sp. and *L. passim*, which parasitise together, has not extensively been investigated. Stevanovic et al. [32] found that the majority of inspected colonies (60.5%) were coinfected with *L. passim* and *N. ceranae* and Vejnovic et al. [33] revealed similar annual dynamics of *L. passim* and *N. ceranae* infection, as well as a seasonality in the occurrence of these parasites: highest burdens of *N. ceranae* and *L. passim* were detected in winter, and lowest in summer—in July, when the temperature was highest. Ravoet et al. [34] reported that the possibility of bee colonies dying in winter is highest when infected with both the trypanosomes and *N. ceranae*, which is why their synergistic negative effect is supposed. 

Owing to the great scarcity of the data on the differences in the prevalence of certain infections, oxidative stress parameters and GOX gene expression between bee colonies kept traditionally and those kept for commercial purposes, this work was aimed at the assessment of (1) oxidative stress parameters (superoxide dismutase (SOD), catalase (CAT), glutathione S-transferase (GST) and malondialdehyde (MDA)), (2) GOX) gene expression and (3) endoparasite (microsporidian and trypanosomatid) prevalence in adult bees in the two types of hives. The comparison of findings obtained in traditional and those in commercial colonies will provide data which can enable better insight into the influence of beekeeping type, i.e., beekeepers’ activities on bee health.

## 2. Materials and Methods 

### 2.1. Sampling and Sample Preparation

**Figure 1:**
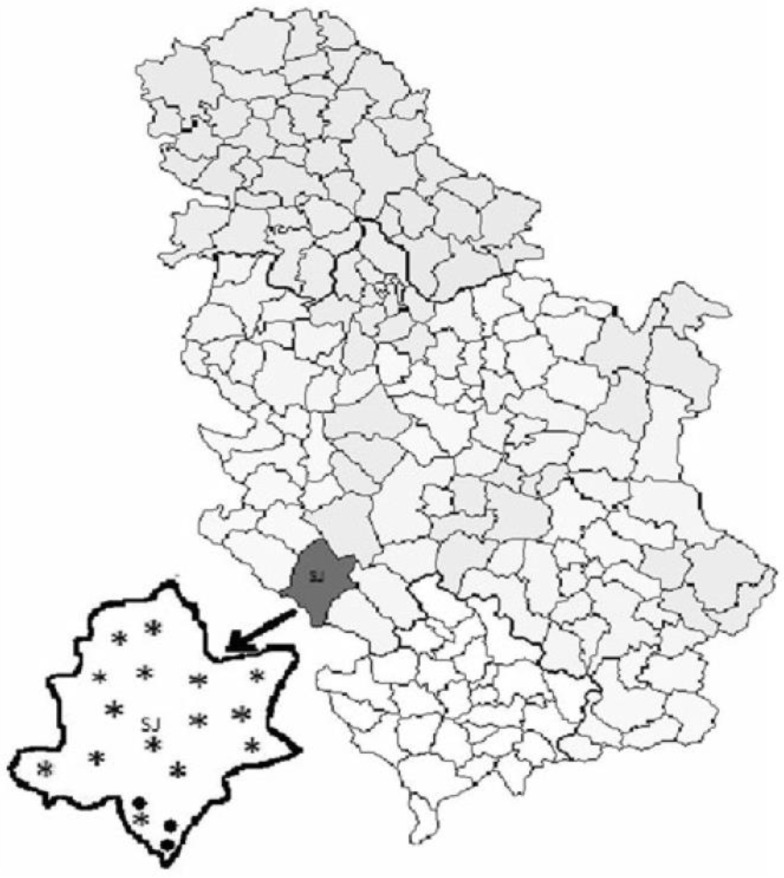
Distribution of the 15 apiaries with commercially kept hives (designated with stars) and 3 apiaries with traditionally kept hives (designated with black dots), from where samples were collected in the Pester plateau (Serbia) [1].

For this study, the same honey bee colonies (both commercial and traditional) as in our previous study [1] were used. At the same location, in a small geographical region called the Pester plateau (Figure 1, published in our previous study [1]), which is located in south-western Serbia (43°16′14′′ N, 19°59′35′′ E), bees were sampled from 15 apiaries with commercially kept colonies (N = 120) in standard DB hives and three consisting of traditionally kept colonies (N = 24) in so-called “trmka” hives. In each apiary, 8 hives were randomly chosen for assessment. In all the 18 apiaries, only stationary beekeeping was practised. According to the beekeepers’ statements commercially kept colonies were subjected to anti-varroa treatments as customary—in August and November. In the region of the Pester plateau, the beekeepers keep 30–50 hives on average. At the end of winter (February–March), the colonies are inspected and the beekeepers take certain apiculture measures accordingly, e.g., provide their bees with various feed (sugar syrup, patties and supplements) and with clean flowing water. In the foraging period (April–August), the major sources of nectar are meadow plants and fruit trees, nothing was added into the bee colonies. All colonies were wintered with enough honey (15–20 kg per colony). The queen bees were purchased and were 1–3 years old. By contrast, traditionally kept colonies had never been provided with additional food, nor treated against varroa infestation, and the royal succession occurred by natural means [1]. They were located in virtually untouched nature, in the environment with ample clean mountain water (streams, wells, ponds and morning dew on plants). The sampling took place in August 2017 (when the colonies were prepared for winter).

Samples of sealed brood (area 10 × 10 cm) were taken from each colony and properly packed [35] to be analysed for the presence of trypanosomatids by PCR method. Samples of adult bees (minimum 140 nurse bees per each hive) were collected with a plastic cup from peripheral brood frames (24 per colony for analyses of oxidative stress parameters, 60 per colony for the analysis of trypanosomatids and microsporidia presence and 50 per colony for the determination of the GOX gene expression level). Every bee sample was divided into three sterile disposable vials, each containing 24, 60 and 50 bee individuals. Bees intended for oxidative stress parameter analysis were frozen in dry ice and processed immediately on arrival at the laboratory. Liquid nitrogen was added to frozen samples, which were then crushed and pulverised in sterile mortars. The Pulver obtained was mixed with a Tris-HCl buffer (10% *w/v*) adjusted to pH 7.4. The homogenates were centrifuged at 10,000× *g* (4 °C) and stored at −80 °C until further analysis. Those sampled for pathogen analysis were also frozen in dry ice, but were stored at −20 °C until processing. Bees intended for GOX gene expression level determination were stored in DNA/RNA Shield (Zymo Research) to protect the gene expression profile.

### 2.2. Oxidative Stress Parameters

Spectrophotometric determination of the activities of antioxidative enzymes superoxide dismutase (SOD), catalase (CAT) and glutathione S-transferase (GST), and the concentrations of malondialdehyde (MDA) were done from pooled samples, each containing 24 bees per colony. The analyses were done on UV/VIS Spectrophotometer BK-36 S390 (Biobase). CAT activity was estimated by using H_2_O_2_ as a substrate following the method by Aebi [36]. One unit of CAT activity was defined as the amount that decomposes 1 mol of H_2_O_2_ and is expressed as U mg^–1^ protein. CAT activity was determined spectrophotometrically at 240 nm, using the extinction coefficient of 43.6 M^−1^ cm^−1^. The GST activity determined according to the method of Habig et al. [37]. GST spectrophotometric assay was monitored by following the conjugation of reduced glutathione to 1-chloro-2,4-dinitrobenzene at 340 nm during 3 min at 25 °C. Enzyme activities were expressed as μmol/min/mg proteins. Superoxide dismutase activity was measured indirectly by monitoring the degree of inhibition of adrenalin auto-oxidation to adrenochrome in alkaline medium at 480 nm [38]. The concentration of MDA was estimated according to the method by Girotti et al. [39], which is based on measurement of the purple colour generated by the reaction of MDA and TBA complex at 530 nm. The results were expressed as nmol of MDA produced per mg protein using molar extinction coefficient 1.56 × 10^−5^/mol/cm. The concentration of protein in homogenates was determined by the Bradford [40] method and BSA was used to construct the calibration curve.

### 2.3. Detection and Species Identification of Trypanosomatids and Microsporidians

In order to detect trypanosomatids (*Lotmaria passim/Crithidia mellificae*) and microsporidians (*Nosema ceranae/apis*), their DNA was extracted with DNeasy Plant Mini Extraction Kit (Qiagen, Hilden, Germany) from pooled samples containing at least 30 pupae per colony (contained in sampled 10 × 10 cm piece of sealed brood) and pooled samples containing 60 adult bees per colony (N = 144 colonies; 120 commercial and 24 traditional). The detection and determination of Nosema species were carried out by a duplex PCR method using primers (Table 1) and the protocol by Martín-Hernández et al. [41]. The 25 μL PCR reaction mixture contained 1× PCR buffer, 0.4 mM of each dNTP, 3 mM MgCl_2_, 0.5 U of Taq polymerase (all by KapaBiosystems), 0.4 μM of each primer (Invitrogen), 0.2 mg/mL bovine serum albumin (New England Biolabs) and 5 μL of template DNA. 

The thermocycler program consisted of 94 °C for 2 min, followed by 10 cycles of 15 s at 94 °C, 30 s at 61.8 °C and 45 s at 72 °C, 20 cycles of 15 s at 94 °C, 30 s at 61.8 °C and 50 s at 72 °C plus an additional 5 s of elongation for each successive cycle, and a final extension step at 72 °C for 7 min. For detection and identification of trypanosome species, primers (Table 1) and PCR protocol by Stevanovic et al. [32] were used. Briefly, PCRs were performed in 20 µL volumes containing 1× PCR buffer, 200 µM dNTP, 0.5 mM MgCl_2_, 0.5 U of Taq polymerase (all produced by KapaBiosystems), 0.3 µM of each primer and 1 µL of template DNA. The PCR parameters were: 2 min at 95 °C, 40 cycles of 30 s at 95 °C, 30 s at 55 °C (for *L. passim*) or 59 °C (for *C. mellificae*) and 20 s at 72 °C, terminated with 2 min at 72 °C.

### 2.4. Glucose Oxidase (GOX) Gene Expression Analysis

From pooled samples containing 50 bees per colony which were kept in DNA/RNA Shield (Zymo Research), the total RNA was isolated using Quick-RNA™ MiniPrep (Zymo Research, Irvine, CA, USA), in accordance with the manufacturer’s instructions. After conversion into cDNA using the FastGene 55-Scriptase cDNA Synthesis set (Nippon Genetics), in compliance with the manufacturer’s instructions, real-time PCR amplification was done using SYBR green method with the KAPA SYBR^®^ FAST qPCR Kit (KAPA Biosystems, Wilmington, MA, USA) in accordance with the manufacturer’s instructions: reaction mixture (20 µL) contained 1× KAPA SYBR FAST qPCR Master Mix (2×) Universal, 200 nM of each primer, 1 μL (5 ng) of cDNA. Primers by Yang and Cox-Foster [42] are given in Table 1. The qPCR reactions were carried out in triplicate in “Rotor-Gene Q 5plex” (Qiagen) using following thermal protocol: 2 min at 95 °C, 40 cycles of amplification with 20 s at 95 °C, 30 s at 60 °C and 80 s at 72 °C. The relative quantification was done with the 2^−ΔCt^ method [43] using β-actin as an internal control gene, for the normalisation of GOX gene expression.

### 2.5. Statistical Analysis

The statistical analysis was performed with GraphPad Prism version 6 (GraphPad, San Diego, CA, USA). The data obtained for SOD, CAT, GST, MDA and GOX were tested for normality using Shapiro–Wilk’s or Kolmogorov–Smirnov’s normality test. Normality was rejected (Shapiro–Wilk’s test, *p* < 0.05; Kolmogorov–Smirnov test, *p* < 0.05), and the differences in the median values of each enzyme between the two bee groups (commercially and traditionally kept) bees were assessed using Mann–Whitney U test.

Fisher’s exact test was used to compare differences in the occurrence of pathogens between bee colonies kept in commercial and in traditional beehives. Significance was estimated at *p* < 0.05 and *p* < 0.01 significance levels.

## 3. Results

**Figure 2:**
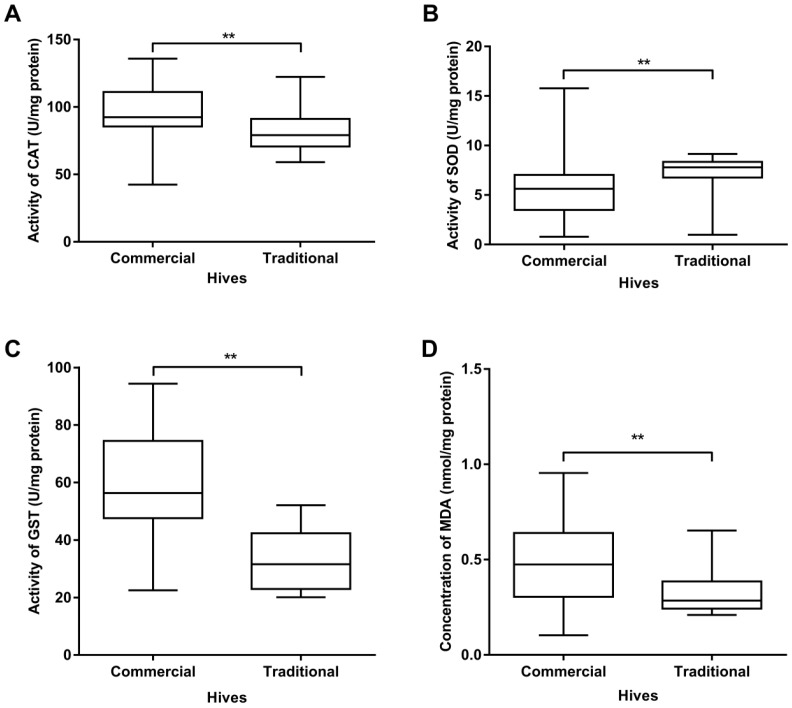
Activities of superoxide dismutase (SOD) (**A**), catalase (CAT) (**B**) and glutathione S-transferase (GST) (**C**) and malondialdehyde (MDA) concentrations (**D**) in adult bees kept in commercial and traditional hives. ** Significant at *p* < 0.01 level.

The analysis of oxidative stress parameters detected significant differences between the two groups of hives—commercially vs. traditionally kept bees. The activities of CAT and GST were significantly higher (*p* < 0.01) in commercially kept colonies (Figure 2A,C). By contrast, the activity of SOD was significantly higher (*p* < 0.01) in traditional hives (Figure 2B). MDA concentration (Figure 2D) also significantly differed between commercially and traditionally kept bees, being significantly higher (*p* = 0.002) in commercial ones. 

**Figure 3:**
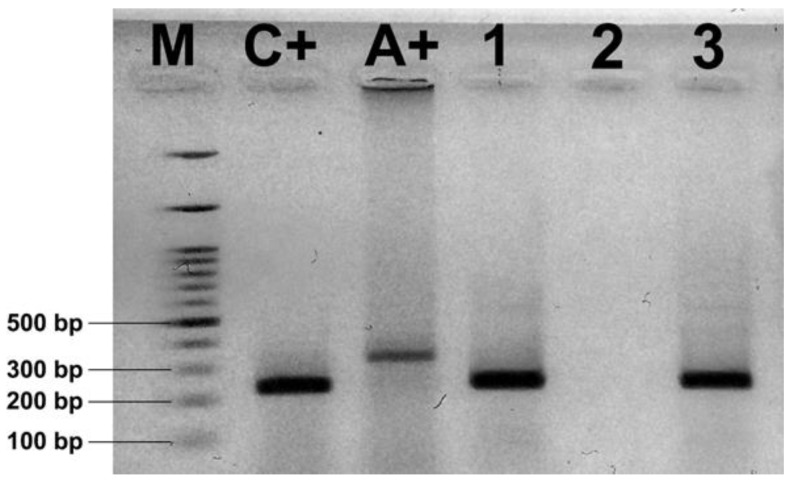
Ethidium-bromide-stained agarose gel showing the results of duplex PCR using primers 321APIS-for/rev and 218MITOC-for/rev for differential diagnostics of *N. ceranae/N. apis*. L—100 bp ladder DNA marker; C+ positive *N. ceranae* control; A+, positive *N. apis* control; 1–3, tested samples.

Duplex PCR revealed the presence of *N. ceranae* (Figure 3) in 61.67% of samples (N = 74) from commercial hives and in 29.17% of samples (N = 7) originating from traditional ones (Table 2). 

**Figure 4:**
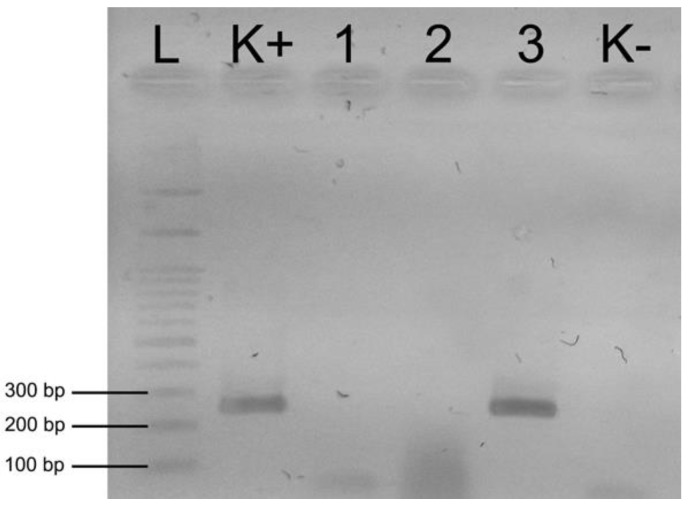
Gel electrophoresis of DNA amplification products obtained with primers LpCytb_F1 and LpCytb_R for detection of *L. passim*. L—100 bp ladder DNA marker; K+, positive *L. passim* control; 1–3, samples; 3, PCR product that correspond to *L. passim*; K–, negative control.

DNA of *L. passim* (Figure 4) was detected in 16.67% of brood samples (N = 20) and 50.00% of adult bee samples (N = 60) from colonies kept for commercial purposes (Table 2). In colonies kept in a traditional way, *L. passim* was confirmed in 8.33% of brood samples (N = 2) and 25% of adult bee samples (N = 6; Table 2). Furthermore, all *L. passim*-positive samples originated from one apiary, which was located in the vicinity of a commercial one. Neither *N. apis* nor *C. mellificae* was found in any sample irrespectively of their origin and sample type. 

In commercial colonies the occurrence of *L. passim* in adult bees 50.00% (N = 60) was significantly higher (*p* < 0.01) than in the brood 16.67% (N = 20). However, in trmka hives between the occurrence of *L. passim* in the samples of bee brood 8.33% (N = 2) and of adult bees 25.00% (N = 6), no significant difference was detected (Table 3). 

**Figure 5:**
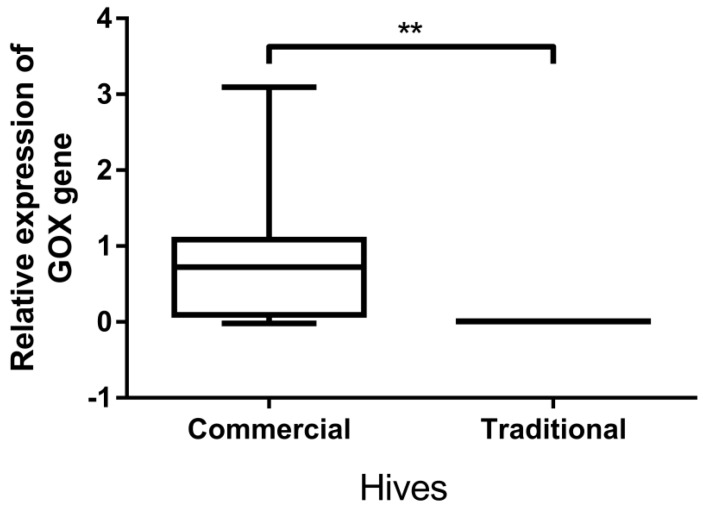
Glucose oxidase (GOX) gene expression levels: comparison between bees originating from commercial hives vs. trmka hives. ** Significant at *p* < 0.01 level.

GOX gene expression level (Figure 5) was significantly (*p* < 0.01) higher in bees originating from commercial hives (median 0.723 (interquartile range (IQR) 0.092–1.087)), than in those kept in trmka hives (median 0.008 (IQR: 0.006–0.009)).

## 4. Discussion

In our research two groups of bee colonies were targeted: (1) those kept for commercial purposes, in which beekeepers apply all standard beekeeping measures (with the exception of migration) in order to achieve maximum productivity, and (2) colonies kept in traditional hives, which were provided only with “natural accommodation”, i.e., trmka hives, and which developed and maintained on their own, without any human influence, not unlike feral bees. The design of the experiment itself enabled the assessment of oxidative stress, endoparasite prevalence and social immunity in these two groups. However, neither the influence of the hives themselves (trmka vs. DB) nor the management system (commercially managed vs. not managed) could have been cross-checked given that the structure of the traditional trmka hive (unmovable honeycombs and the missing possibility of opening the hive), as well as the organisation of the bee colony, does not enable any of the procedures of bee management which is characteristic of commercial hives and any manipulation would lead to the killing of the colony in a trmka hive. Undoubtedly, these two factors can exert a significant influence on the health status of bees. In future, it is necessary to find out a method which can enable the research into the influence of either of the factors taken separately. This research is a sequel to a previous scientific work [1] conducted in order to get a better insight into the problem of commercial bee colony losses recently noticed worldwide [44,45].

The results showed that commercial and traditional colonies significantly differ in the parameters of oxidative stress (SOD, CAT, GST and MDA), as well as in the prevalence of *N. ceranae* and *L. passim* (Figure 2, Table 2 and Table 3) infections. 

The results of our research revealed significantly higher activities of SOD in traditionally kept bees in comparison to commercial colonies. It is known that SOD catalyses the dismutation of superoxide radical (O^2−^) to hydrogen peroxide (H_2_O_2_) and is the first-line defence against ROS [46]. 

By contrast, in our research significantly higher activity of CAT was measured in commercial colonies than in traditional ones. This was possibly the reflection of bees’ response to higher parasite burdens (microsporidia and trypanosomes) in commercial colonies, as it was previously detected that CAT is part of the immune system and has an important protective role in insects infected with intestinal parasites [47].

In commercial colonies, a significantly higher GST activity was measured than in traditionally kept colonies, probably due to higher pathogen burdens, which is in accordance with the results obtained by Vidau et al. [48] and Dussaubat et al. [10]. Given that both microsporidia and trypanosomes are gut parasites, the explanation for this phenomenon proposed by Dubovskiy et al. [8] can be accepted: it claimed that GST is involved in the inactivation of toxic products of lipid peroxidation that accumulate during gut destruction brought about by these parasites. This is also in line with our results of MDA analysis, given that this marker of lipid peroxidation in the current work was significantly higher in commercially kept colonies probably owing to increased lipid peroxidation caused by substantially higher pathogen prevalence. Some other authors [8,49,50] have also pointed to increased lipid peroxidation due to the presence of pathogens in insects. Thus, it is possible that significantly higher values of oxidative stress parameters (CAT, GST and MDA) in commercial colonies resulted from higher burdens of parasites. We presume that the cause of both increased parasite prevalence and stress in commercial colonies is the anthropogenic influence, that is, inadequate beekeeping practices [12]. This is in line with some previous findings that imbalanced food and different metabolic demands develop oxidative and energetic stress [23,51,52,53,54]. Significantly higher GOX gene expression in commercially kept colonies than in those kept in trmka hives is an additional finding in the current research. All colonies, both traditionally and commercially kept, have been situated on the Pester plateau, live in similar environmental conditions and feed on the same melliferous flora but commercially kept colonies are overexploited, fed on sugar (given in conditions of scarce natural forage and for queen forcing) and treated with anti-varroa substances and other, often unregistered preparations. Although these facts may indicate the beekeeping practice as the reason of worse condition we documented in commercial vs. traditional hives, we should not exclude different genetic background of the colonies that might have significantly influenced honey bee health, including immunocompetence and gene expression, as was already shown by López-Uribe et al. [19,55].

Research into bee brood in our previous work [1] revealed significantly higher pathogen prevalence (*Paenibacillus larvae*, *Melissococcus plutonius*, *Ascosphaera apis* and sacbrood virus—SBV) in colonies kept for commercial purposes in comparison to their traditional counterparts. In this work, in commercially kept colonies higher prevalence of *L. passim* (in brood and in adult bees) and *N. ceranae* (in adult bees) was discovered, but also a significantly higher mRNA level for GOX. 

In contrast to commercially kept colonies, the bees in trmka hives were significantly less burdened with pathogens. Besides, the influence of beekeepers on these was substantially weaker: they were not given unnatural food (syrups and patties made by beekeepers), were not deprived of honey and were not treated with veterinary preparations. Rather, they had ample self-made, balanced, natural food (honey and bee bread) and thus did not need to increase GOX synthesis, probably owing to established homeostasis as a result of the absence of disturbing effects of beekeepers (beekeeping procedures), which are inevitable and common in commercial colonies (“sugarisation”, unprofessional acaricide use and repeated hive inspection, which all disturb bees). Pure sugar (in the form of syrup or patties), which is frequently given to bees kept for commercial purposes, may induce energetic and oxidative stress, the expansion of pathogens, exhaustion of bees, their increased mortality and, frequently, total colony collapse [12].

Bee colonies are frequently apparently healthy—various pathogens may long be present in bees not causing clinical symptoms [1,56,57]. Epidemiological research revealed a high prevalence of nosemosis in commercial apiaries worldwide [32,58,59,60,61]. In those colonies, imbalanced diet in combination with other stressors (pathogens and parasites, agropesticides, beekeepers’ manipulations) is a “factor plus”, which leads to weakening of the colony, immunity suppression, disturbance of the host–parasite relationship, energetic and oxidative stress, and, eventually, to the disruption of colonies [12,22,62]. In addition to this, chemisation of the environment with agropesticides and excessive administration of acaricides to commercial hives negatively affect bee health, immunity and behaviour [63,64,65] and spoils the quality of bee products [66,67,68,69,70]. In the long term, the use of anti-varroa products disturbs the host–parasite co-evolution [71].

Significantly (*p* < 0.01) higher prevalence of *N. ceranae* infection was detected in samples taken from commercial hives (61.67%) in comparison to those from traditional colonies (29.17%), but it may only be assumed that the reason for this was the limited exposure of the latter to anthropogenic influence, i.e., to beekeepers’ practices (queen replacement and rearing, manipulation with honeycombs and honeycomb foundations, inadequate feeding and increased disturbance of commercial hives owing to frequent, usually unnecessary opening of hives and bee inspection), since they encourage the spread of pathogens. 

In this study, *L. passim* was detected not only in adult bees, but also in the brood in both commercial and traditional colonies. The prevalence of *L. passim* in adult bees (in 50% of commercial and 25% of traditional colonies) is rather higher than in the USA (16% of commercial and 4% of feral colonies), according to Williams et al. [5], but significantly lower than in Chile (90%), as claimed by Arismendi et al. [30]. The prevalence of infection in adult bees in commercial colonies (50%) is similar to the value obtained in previous research in Serbia (60%), when samples collected in the period from 2007 to 2015 were retrospectively analysed [32]. As for the presence of *L. passim* in bee brood, this is the first detection of DNA of *L. passim* in bee brood, but further investigations are necessary to determine whether this trypanosome parasitises bee larvae. However, the prevalence of *L. passim* is higher in adult bees than in larvae (in commercial colonies even significantly higher, *p* < 0.01). Significantly (*p* < 0.05) lower prevalence of *L. passim* in colonies kept traditionally in comparison with those kept for commercial purposes probably results from the absence of beekeeping measures in the former, which can facilitate the spread of infections. Similar results were obtained by Williams et al. [5], who compared commercial and feral bee colonies and detected significantly lower prevalence of *L. passim* in the latter. In our research, *L. passim* was found in trmka hives in only one apiary, which was located in the vicinity of a commercial one, from which the infection might have spread through the shared use of flowers, as was evidenced for another trypanosomatid parasite, *Crithidia bombi* [72]. 

Our results of the high prevalence of *L. passim* (50.00% in adult bees and 16.00% in brood) and *N. ceranae* (61.67%) in commercial colonies are in line with those obtained by Stevanovic et al. [32], who diagnosed co-infections with both parasites in 60.5% of colonies surveyed over the nine-year period (2005–2017). These results suggest that there is a possibility of connection existing between the two gut parasites, *L. passim* and *N. ceranae*, but hitherto only a significant positive correlation between their infection levels, similar annual dynamics and seasonality has been detected [33]. Given that it is now a mere hypothesis, it is yet to be determined if they act synergistically on bees, having in mind the possibility that colonies succumb to winter losses is higher when coinfected with *N. ceranae* and trypanosomes [31].

## 5. Conclusions

In summary, in comparison to traditionally kept colonies, commercially kept ones had higher oxidative stress and higher prevalence of *N. ceranae* and *L. passim*. However, to decide if it is a reflection of their reaction to stressors and efforts to mitigate the negative anthropogenic factors, more detailed studies are needed.

## Figures and Tables

**Table 1:** Primers used in this study.

Primer	Target	Sequence (5′–3′)	Reference
218MITOC-for 218MITOC-rev	*Nosema ceranae*	CGGCGACGATGTGATATGAAAATATTAA CCCGGTCATTCTCAAACAAAAAACCG	[41]
321APIS-for 321APIS-rev	*N. apis*	GGGGGCATGTCTTTGACGTACTATGTA GGGGGGCGTTTAAAATGTGAAACAACTATG	
LpCytb_F1 LpCytb_R	*Lotmaria passim*	cGAAGTgCaCATATATGCTTtAC gcCAaAcACCaATaACtGGtACt	[32]
CmCytb_F CmCytb_R	*Crithidia mellificae*	AGTtTGAgCtGTtGGaTTTgTt AACCtATtACaGGcACaGTTGC	
GOX_F GOX_R	Glucose oxidase (GOX)	GAGCGAGGTTTCGAATTGGA GTCGTTCCCCCGAGATTCTT	[42]

**Table 2:** Prevalence of *N. ceranae* and *L. passim* in commercially and traditionally kept bee colonies based on PCR detection of the parasites’ DNA.

Bee Parasite	Samples	Hives		*Significance*
		Commercial % (N)	Traditional—Trmka % (N)	
*N. ceranae*	Adult bees	61.67 (74)	29.17 (7)	**
*L. passim*	Bee brood	16.67 (20)	8.33 (2)	ns
*L. passim*	Adult bees	50.00 (60)	25.00 (6)	*

** Significant at *p* < 0.01, * *p* < 0.05, ns *p* > 0.05 level.

**Table 3:** Comparison of prevalence of *L. passim* in larvae and adult bees in commercial and traditional hives (trmka hives).

Samples Positive for *L. passim* in Commercial Apiaries—DB Hives			Samples Positive for *L. passim* in Traditional Apiaries—Trmka Hives		
Bee Brood % (N)	Adult Bees % (N)	Significance	Bee Brood % (N)	Adult Bees % (N)	Significance
16.67 (20)	50.00 (60)	**	8.33 (2)	25.00 (6)	ns

** Significant at *p* < 0.01, ns *p* > 0.05 level.

## References

[1] Taric E., Glavinic U., Stevanovic J., Vejnovic B., Aleksic N., Dimitrijevic V., Stanimirovic Z. (2019). Occurrence of honey bee (*Apis mellifera* L.) pathogens in commercial and traditional hives. J. Apicult. Res..

[2] Thompson C.E., Biesmeijer C.J., Allnutt T.R., Pietravalle S., Budge G.E. (2014). Parasite pressures on feral honey bees (*Apis mellifera* sp.). PLoS ONE.

[3] Appler R.H., Frank S.D., Tarpy D.R. (2015). Within-Colony Variation in the Immunocompetency of Managed and Feral Honey Bees (*Apis mellifera* L.) in Different Urban Landscapes. Insects.

[4] Youngsteadt E., Appler R.H., López-Uribe M.M., Tarpy D.R., Frank S.D. (2015). Urbanization Increases Pathogen Pressure on Feral and Managed Honey Bees. PLoS ONE.

[5] Williams M.K.F., Tripodi A.D., Szalanski A.L. (2019). Molecular survey for the honey bee (*Apis mellifera* L.) trypanosome parasites *Crithidia mellificae* and *Lotmaria passim*. J. Apicult. Res..

[6] Sorci G., Faivre B. (2009). Inflammation and oxidative stress in vertebrate host–parasite systems. Philos. T. Roy. Soc. B..

[7] Halliwell B., Gutteridge J.M. (2015). Free radicals in biology and medicine.

[8] Dubovskiy I.M., Martemyanov V.V., Vorontsova Y.L., Rantala M.J., Gryzanova E.V., Glupov V.V. (2008). Effect of bacterial infection on antioxidant activity and lipid peroxidation in the midgut of *Galleria mellonella L. larvae* (Lepidoptera, Pyralidae). Comp. Biochem. Phys. C..

[9] Gülmez Y., Dursun K., Ilyas C. (2016). Effects of *Varroa destructor* Anderson & Trueman Infestation on Antioxidant Enzymes of Adult Worker Honey Bee (*Apis mellifera* L.). Asian. J. Chem..

[10] Dussaubat C., Brunet J.L., Higes M., Colbourne J.K., Lopez J., Choi J.H., Hernández R.M., Botías C., Cousin M., McDonnell C. (2012). Gut pathology and responses to the microsporidium *Nosema ceranae* in the honey bee *Apis mellifera*. PLoS ONE.

[11] Alaux C., Ducloz F., Crauser D., Le Conte Y. (2010). Diet effects on honeybee immunocompetence. Biol. Lett..

[12] Stanimirovic Z., Glavinic U., Ristanic M., Aleksic A., Jovanovic N., Vejnovic B., Stevanović J. (2019). Looking for the causes of and solutions to the issue of honey bee colony losses. Acta Vet..

[13] Wilson-Rich N., Spivak M., Fefferman N.H., Starks P.T. (2009). Genetic, individual group facilitation of disease resistance in insect societies. Annu. Rev. Entomol..

[14] Alaux C., Brunet J.L., Dussaubat C., Mondet F., Tchamitchan S., Cousin M., Brillard J., Baldy A., Belzunces L.P., Le Conte Y. (2010). Interactions between *Nosema* microspores and a neonicotinoid weaken honeybees (*Apis mellifera*). Environ. Microbiol..

[15] Jones B., Shipley E., Arnold K.E. (2018). Social immunity in honeybees—Density dependence, diet, and body mass trade-offs. Ecol. Evol..

[16] Visscher P.K. (1980). Adaptations of honey bees (*Apis mellifera*) to problems of nest hygiene. Sociobiology.

[17] Sano O., Kunikata T., Kohno K., Iwaki K., Ikeda M., Kurimoto M. (2004). Characterization of royal jelly proteins in both Africanized and European honeybees (*Apis mellifera*) by two-dimensional gel electrophoresis. J. Agric. Food. Chem..

[18] Brudzynski K. (2006). Effect of hydrogen peroxide on antibacterial activities of Canadian honeys. Can. J. Microbiol..

[19] López-Uribe M.M., Fitzgerald A., Simone-Finstrom M. (2017). Inducible versus constitutive social immunity: Examining effects of colony infection on glucose oxidase and defensin-1 production in honeybees. Roy. Soc. Open. Sci..

[20] Fries I. (2010). Nosema ceranae in European honey bees (*Apis mellifera*). J. Invertebr. Pathol..

[21] Higes M., Martín-Hernández R., Meana A. (2010). *Nosema ceranae* in Europe: An emergent type C nosemosis. Apidologie..

[22] Martín-Hernández R., Botías C., Barrios L., Martínez-Salvador A., Meana A., Mayack C., Higes M. (2011). Comparison of the energetic stress associated with experimental *Nosema ceranae* and *Nosema apis* infection of honeybees (*Apis mellifera*). Parasitol. Res..

[23] Mayack C., Naug D. (2009). Energetic stress in the honeybee *Apis mellifera* from *Nosema ceranae* infection. J. Invertebr. Pathol..

[24] Antúnez K., Martín-Hernández R., Prieto L., Meana A., Zunino P., Higes M. (2009). Immune suppression in the honey bee (*Apis mellifera*) following infection by *Nosema ceranae* (Microsporidia). Environ. Microbiol..

[25] Chaimanee V., Chantawannakul P., Chen Y., Evans J.D., Pettis J.S. (2012). Differential expression of immune genes of adult honey bee (*Apis mellifera*) after inoculated by *Nosema ceranae*. J. Insect Physiol..

[26] Glavinic U., Stankovic B., Draskovic V., Stevanovic J., Petrovic T., Lakic N., Stanimirovic Z. (2017). Dietary amino acid and vitamin complex protects honey bee from immunosuppression caused by *Nosema ceranae*. PLoS ONE.

[27] Goblirsch M., Huang Z.Y., Spivak M. (2013). Physiological and behavioral changes in honey bees (*Apis mellifera*) induced by *Nosema ceranae* infection. PLoS ONE.

[28] Higes M., Martín R., Meana A. (2006). *Nosema ceranae*, a new microsporidian parasite in honeybees in Europe. J. Invertebr. Pathol..

[29] Schwarz R.S., Bauchan G.R., Murphy C., Ravoet J., de Graaf D.C., Evans J.D. (2015). Characterization of two species of Trypanosomatidae from the honey bee *Apis mellifera*: *Crithidia mellificae* Langridge and McGhee, and *Lotmaria passim*. J. Eukaryot. Microbiol..

[30] Arismendi N., Bruna A., Zapata N., Vargas M. (2016). PCR-specific detection of recently described *Lotmaria passim* (Trypanosomatidae) in Chilean apiaries. J. Invertebr. Pathol..

[31] Ravoet J., Schwarz R.S., Descamps T., Yañez O., Tozkar C.O., Martín-Hernández R., Bartolomé C., De Smet L., Higes M., Wenseleers T. (2015). Differential diagnosis of the honey bee trypanosomatids *Crithidia mellificae* and *Lotmaria passim*. J. Invertebr. Pathol..

[32] Stevanovic J., Schwarz R.S., Vejnovic B., Evans J.D., Irwin R.E., Glavinic U., Stanimirovic Z. (2016). Species-specific diagnostics of *Apis mellifera* trypanosomatids: A nine-year survey (2007–2015) for trypanosomatids and microsporidians in Serbian honey bees. J. Invertebr. Pathol..

[33] Vejnovic B., Stevanovic J., Schwarz R.S., Aleksic N., Mirilovic M., Jovanovic N., Stanimirovic Z. (2018). Quantitative PCR assessment of *Lotmaria passim* in *Apis mellifera* colonies co-infected naturally with *Nosema ceranae*. J. Invertebr. Pathol..

[34] Ravoet J., Maharramov J., Meeus I., de Smet L., Wenseleers T., Smagghe G., de Graaf D.C. (2013). Comprehensive bee pathogen screening in Belgium reveals *Crithidia mellificae* as a new contributory factor to winter mortality. PLoS ONE.

[35] OIE-Office International Des Epizooties (2019). Manual of Diagnostic Tests and Vaccines for Terrestrial Animals. http://www.oie.int/en/international-standard-setting/terrestrial-manual/access-online/.

[36] Aebi H. (1984). Catalase in vitro. Packer Lester. 1st ed: Methods Enzymol.

[37] Habig W.H., Pabst M.J., Jakoby W.B. (1974). Glutathione S-transferases the first enzymatic step in mercapturic acid formation. J. Biol. Chem..

[38] Misra H.P., Fridovich I. (1972). The role of superoxide anion in the autoxidation of epinephrine and a simple assay for superoxide dismutase. J. Biol. Chem..

[39] Girotti M.J., Khan N., McLellan B.A. (1991). Early measurement of systemic lipid peroxidation products in the plasma of major blunt trauma patients. J. Trauma. Acute. Care..

[40] Bradford M.M. (1976). A rapid and sensitive method for the quantitation of microgram quantities of protein utilizing the principle of protein dye binding. Anal. Biochem..

[41] Martín-Hernández R., Meana A., Prieto L., Salvador A.M., Garrido-Bailón E., Higes M. (2007). Outcome of colonization of *Apis mellifera* by *Nosema ceranae*. Appl. Environ. Microbiol..

[42] Yang X., Cox-Foster D.L. (2005). Impact of an ectoparasite on the immunity and pathology of an invertebrate: Evidence for host immunosuppression and viral amplification. Proc. Natl. Acad. Sci. USA.

[43] Evans J.D. (2006). Beepath: An ordered quantitative-PCR array for exploring honey bee immunity and disease. J. Invertebr. Pathol..

[44] Brodschneider R., Gray A., Adjlane N., Ballis A., Brusbardis V., Charrière J.D., Chlebo R., Coffey M.F., Dahle B., de Graaf D.C. (2018). Multi-country loss rates of honey bee colonies during winter 2016/2017 from the COLOSS survey. J. Apicult. Res..

[45] Grey A., Brodschneider R., Adjlane N., Ballis A., Brusbardis V., Charrière J.D., Chlebo R., Coffey M.F., Cornelissen B., da Costa C.A. (2019). Loss rates of honey bee colonies during winter 2017/18 in 36 countries participating in the COLOSS survey, including effects of forage sources. J. Apicult. Res..

[46] Surai P.F. (2015). Antioxidant Systems in Poultry Biology: Superoxide Dismutase. J. Anim. Res. Nut..

[47] Ha E.M., Oh C.T., Ryu J.H., Bae Y.S., Kang S.W., Jang I.H., Brey P.T., Lee W.J. (2005). An antioxidant system required for host protection against gut infection in *Drosophila*. Dev. Cell..

[48] Vidau C., Diogon M., Aufauvre J., Fontbonne R., Viguès B., Brunet J.L., Texier C., Biron D.G., Blot N., Alaoui H.E. (2011). Exposure to sublethal doses of fipronil and thiacloprid highly increases mortality of honeybees previously infected by *Nosema ceranae*. PLoS ONE.

[49] Ahmed A.M. (2012). Lipid peroxidation and oxidative protein products as biomarkers of oxidative stress in the autogenous mosquito, *Aedes caspius*, upon infection with the mosquitocidal bacterium, *Bacillus thuringiensis kurstaki*. Pakistan. J. Zool..

[50] Wang Y., Oberley L.W., Murhammer D.W. (2001). Evidence of oxidative stress following the viral infection of two Lepidopteran insect cell lines. Free. Rad. Biol. Med..

[51] Nikolić V.T., Purac J., Orcic S., Kojic D., Vujanovic D., Stanimirovic Z., Grzetic I., Ilijevic K., Sikoparija B., Blagojevic P.D. (2015). Environmental effects on superoxide dismutase and catalase activity and expression in honey bee. Arch. Insect Biochem..

[52] Orcic S., Nikolic T., Purac J., Sikoparija B., Blagojević P.D., Vukasinovic E., Plavsa N., Stevanovic J., Kojic D. (2017). Seasonal variations in the activity of selected antioxidant enzymes and malondialdehyde level in worker honey bees. Entomol. Exp. Appl..

[53] Simone-Finstrom M., Li-Byarlay H., Huang M.H., Strand M.K., Rueppell O., Tarpy D.R. (2016). Migratory management and environmental conditions affect lifespan and oxidative stress in honey bees. Sci. Rep..

[54] Glavinic U. (2019). The effects of various antimicrobials and supplements on the expression of immune-related genes, oxidative stress and survival of honey bee *Apis mellifera* infected with microsporidium *Nosema ceranae*. Ph.D. Thesis.

[55] López-Uribe M.M., Appler R.H., Youngsteadt E., Dunn R.R., Frank S.D., Tarpy D.R. (2017). Higher immunocompetences associated with higher genetic diversity in feral honey bee colonies (*Apis mellifera*). Conserv. Genet..

[56] Simeunovic P. (2015). Molecular detection and identification of microsporidia and viruses in honey bee colonies in Serbia. Ph.D. Thesis.

[57] Cirkovic D., Stevanovic J., Glavinic U., Aleksic N., Djuric S., Aleksic J., Stanimirovic Z. (2018). Honeybee viruses in Serbian colonies of different strength. PeerJ.

[58] Fernández J.M., Puerta F., Cousinou M., Dios-Palomares R., Campano F., Redondo L. (2012). Asymptomatic presence of *Nosema* spp. in Spanish commercial apiaries. J. Invertebr. Pathol..

[59] Stevanovic J., Stanimirovic Z., Genersch E., Kovacevic R.S., Ljubenkovic J., Radakovic M., Aleksic N. (2011). Dominance of *Nosema ceranae* in honey bees in the Balkan countries in the absence of symptoms of colony collapse disorder. Apidologie.

[60] Stevanovic J., Simeunovic P., Gajic B., Lakic N., Radovic D., Fries I., Stanimirovic Z. (2013). Characteristics of *Nosemaceranae* infection in Serbian honey bee colonies. Apidologie.

[61] Traver B.E., Fell R.D. (2011). Prevalence and infection intensity of Nosema in honey bee (*Apis mellifera* L.) colonies in Virginia. J. Invertebr. Pathol..

[62] Bordier C., Suchail S., Pioz M., Devaud J.M., Collet C., Charreton M., Le Conte Y., Alaux C. (2017). Stress response in honeybees is associated with changes in task-related physiology and energetic metabolism. J. Insect Physiol..

[63] Kiljanek T., Niewiadowska A., Posyniak A. (2016). Pesticide poisoning of honeybees: A review of symptoms, incident classification, and causes of poisoning. J. Apic. Sci..

[64] Sánchez-Bayo F., Goulson D., Pennacchio F., Nazzi F., Goka K., Desneux N. (2016). Are bee diseases linked to pesticides?—A brief review. Environ. Int..

[65] Glavinic U., Tesovnik T., Stevanovic J., Zorc M., Cizelj I., Stanimirovic Z., Narat M. (2019). Response of adult honey bees treated in larval stage with prochloraz to infection with *Nosema ceranae*. PeerJ.

[66] Stanimirovic Z., Pejovic D., Stevanovic J., Vucinic M., Mirilovic M. (2002). Investigations of hygienic behaviour and disease resistance in organic beekeeping of two honeybee ecogeographic varieties from Serbia. Acta Vet..

[67] Stanimirovic Z., Stevanovic J., Cirkovic D. (2005). Behavioural defenses of the honey bee ecotype from Sjenica–Pester against *Varroa destructor*. Acta Vet..

[68] Stanimirovic Z., Stevanovic J., Jovanovic S., Andjelkovic M. (2005). Evaluation of genotoxic effects of Apitol^®^ (cymiazole hydrochloride) in vitro by measurement of sister chromatid exchange. Mutat. Res. Genet. Toxicol. Environ. Mutagen..

[69] Stanimirovic Z., Stevanovic J., Bajic V., Radovic I. (2007). Evaluation of genotoxic effects of fumagillin by cytogenetic tests in vivo. Mutat. Res. Genet. Toxicol. Environ. Mutagen..

[70] Stevanovic J., Stanimirovic Z., Radakovic M., Stojic V. (2008). In vitro evaluation of the clastogenicity of fumagillin. Environ. Mol. Mutagen..

[71] Neumann P., Blacquière T. (2017). The Darwin cure for apiculture? Natural selection and managed honeybee health. Evol. Appl..

[72] Ruiz-González M.X., Brown M.J. (2006). Honey bee and bumblebee trypanosomatids: Specificity and potential for transmission. Ecol. Entomol..

